# Changes in bacterial community composition and antibiotic resistance profiles of coral-associated microbiota in the vicinity of Chilean salmon farms

**DOI:** 10.1038/s41598-026-57517-y

**Published:** 2026-06-20

**Authors:** Anna Berezkina, Tanja Rahn, Mailin Suchantke, Kristina K. Beck, Juan Pablo Espinoza, Claudio Richter, Ute Hentschel, Marlene Wall

**Affiliations:** 1 Alfred Wegener Institute Helmholtz Centre for Polar and Marine Research, Bremerhaven, Germany; 2 GEOMAR Helmholtz Centre for Ocean Research, Kiel, Germany; 3 University of Edinburgh, Edinburgh, UK; 4 Fundación San Ignacio del Huinay, Puerto Montt, Chile; 5 University of Bremen, Bremen, Germany

**Keywords:** Cold-water corals, Aquaculture activity, Salmon farming, Microbial dynamics, Antibiotic resistance, Temperate fjords, Ecology, Ecology, Microbiology

## Abstract

The extensive use of antibiotics in Chilean salmon farming raises concerns about harmful effects on benthic habitats, particularly the iconic cold-water coral (CWC) banks in semi-enclosed fjords. We collected specimens of the CWC *Desmophyllum dianthus* near and far from salmon farms in Comau Fjord, Chile, and investigated their associated bacterial community (metabarcoding, culturing) and screened bacterial isolates for their antibiotic resistance (15 antibiotics, including antibiotics common in Chilean salmon farming). The cultured bacterial isolates of *D. dianthus* showed a slight but significant increase in antibiotic resistance (near: 7.7 ± 0.47 vs. far: 6.3 ± 0.48). This was accompanied by a change in dominance in the randomly selected isolates, with *Pseudoalteromonas* and *Shewanella* dominating far and near, respectively. Metabarcoding also showed a shift in the dominant bacterial strains from SUP05 clade to *Mycoplasma* and a loss of *Pseudoalteromonas* strains near the salmon farm. Common antibiotics used in salmon farming, however, significantly reduced bacterial growth at the community level, and most bacterial isolates were also sensitive to these antibiotics, except for tilmicosin. This may provide a first indication of how salmon farms modify the microbiome of *D. dianthus* and underscores the need to expand such assessments in space and time.

**Supplementary Information:**

The online version contains supplementary material available at 10.1038/s41598-026-57517-y.

## Introduction

Anthropogenic threats to marine ecosystems raise significant concerns, especially in recent decades, when benthic communities are being heavily impacted by human activities on local, regional and global scale^1^. Among the key drivers are fishing pressure (commercial bottom trawling), deep-sea mining, pollution, eutrophication, and intense aquaculture, such as salmon farming^2,3^. As a result, many marine ecosystems across the world, from shallow waters to the deep ocean, are deteriorating^4^.

Coastal waters are one of the most anthropogenically affected marine areas. Coastal aquaculture, which provides food to a growing human population, has increased significantly over the last decade and is expected to continue to expand over the next 20 years^5^. A notable portion of aquaculture takes place in temperate regions, where it is a critical industry for the producing countries. For example, salmon farming in fjords is of major economic revenue to countries like Norway, Chile, the United Kingdom, Canada, and the Faroe Islands^6,7^. While the aquaculture of salmonids provides important economic returns, its advance into remote pristine habitats is a growing ecological concern, due to the introduction of aquaculture feed and chemicals into the environment^8^. Organic and inorganic materials such as faeces, feed waste, pesticides, antifouling paints, medications and disinfectants have been documented to be released in significant quantities, causing eutrophication and noxious plankton blooms, among others, with negative effects on benthic organisms^9,10^. Dense concentrations of cultured salmon are often accompanied by excessive use of antibiotics, jeopardizing the aquatic environment, increasing the risk of inducing pathogens’ resistance to antibiotics. Environmental bacteria serve as reservoirs of antibiotic resistance genes and a putative exchange of such genes, through mobile genetic elements, between environmental bacteria and human pathogens is to date an under-appreciated route of transmission with potentially severe concerns for human health^8,11–13^.

In the Patagonian fjords of Chile, intense salmon farming activity is accompanied by the use of very high concentrations of antibiotics compared to international standards. In 2019, Chile used 334.1 tons of antibiotics to produce 989,500 tons of salmon. This is over 2000 times more than in Norway, which used 0.201 tons of antibiotics in 2016 to produce 1.3 million tons of farmed seafood (95% of which was Atlantic salmon)^14^. The uncontrolled release of antimicrobial compounds into the environment can change natural microbial populations and induce resistance among environmental bacteria^15^. A previous study in Chile has shown that bacteria from sediments close to salmon farms in the Calbuco Archipelago display resistance to florfenicol, oxytetracycline, and oxolinic acid^16^. Other studies have demonstrated antibiotic resistance to beta-lactams and tetracyclines, bacitracin, and the group macrolide–lincosamide–streptogramin in seawater microbial communities in Comau Fjord, Chile, underscoring the potential consequences for microbial communities^17^. By contrast, little is known about the impact of salmon farms on benthic communities. Only recently, a study in Norway revealed negative effects of salmon farming on metabolic rates of the cold-water coral (CWC) *Lophelia pertusa*^18^ (note that *L. pertusa* was renamed *D. pertusum*^19^, emphasizing potentially far-reaching consequences. Aquaculture in the fjords and channels of Patagonia has experienced sustained growth over the past four decades, but studies documenting the impact of these commercial activities on the natural environment and marine fauna are still in their infancy.

The CWC *Desmophyllum dianthus* is one of the most conspicuous benthic organisms in southern Chilean fjords. This species forms dense populations on rocky substrate^20^, providing refuge for numerous marine fish species and enhancing local biodiversity^21^. Strong phenotypic plasticity of *D. dianthus* was observed in Comau Fjord with compromised health in shallow water populations subjected to high environmental variability^22,23^, and other potential local drivers including salmon farming^14^ may also play a role. Shallow phenotypes were, in general, smaller in size compared to their deep counterparts, though one population near a salmon farm also showed loss of tissue area compared to corals from far-away shallow sites^22^. Cold-water corals are known to be associated with diverse microbial communities, in particular members of the phylum Proteobacteria (Pseudomonata), Bacteroidetes (Bacteroidota), and Firmicutes (Bacillota). The complex association of the coral host with its associated microorganisms is commonly referred to as the coral holobiont. Members of the holobiont can provide important functions to the coral hosts, like nutrient cycling, chemoautotrophy, or antibiotic production. They also allow them to dynamically respond to environmental changes and maintain good health and performance^24,25^. By contrast, changes in microbial associations have been associated with dysbiosis and compromised health^26,27^. As suspension feeders, CWCs like *D. dianthus* may accumulate particulate substances from the marine environment and serve as sentinels of environmental change when their microbiomes shift away from their baselines^26,27^.

The aim of this study was to investigate the impact of salmon farming on CWC and their microbiome, i.e., the holobionts. We assume that the acquisition of antibiotic resistance by bacteria serves as an indicator for the release of large quantities of antibiotics into the marine environment. Specifically, we aimed to explore shifts in culturable microbial composition along with the development of antimicrobial resistance in the CWC *D. dianthus*. We aimed to address the following questions: Do microbial associations change in relation to coral phenotype collected near and far from salmon farms? Have salmon farms contributed to the rise of antibiotic-resistant microbial communities of CWC-associated bacteria? The present study seeks to gain a deeper understanding of the aquaculture influence on the microbial associations of a CWC benthic foundation species.

## Results

### Antibiotic resistance of coral-associated bacteria

**Fig. 1:**
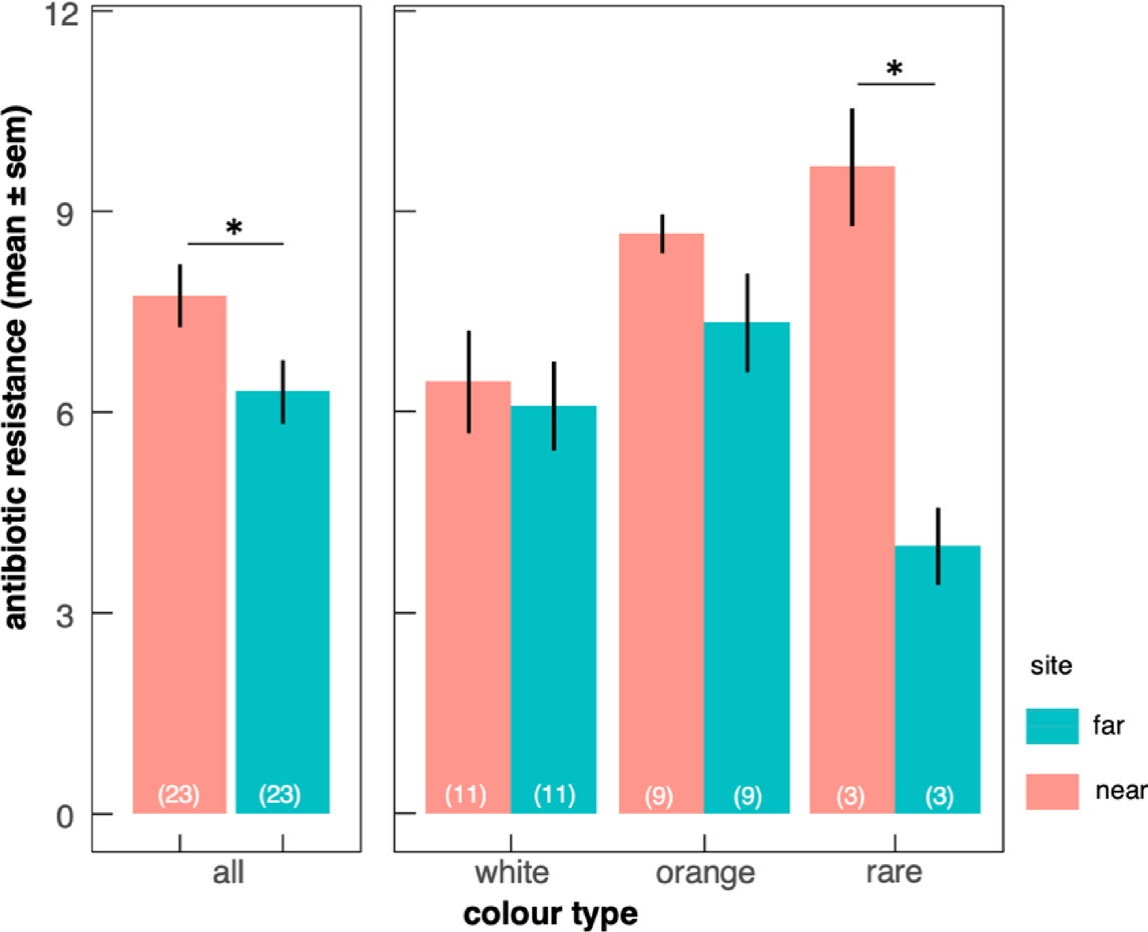
Antibiotic resistances of bacterial isolates associated with the cold-water coral *Desmophyllum dianthus*. Antibiotic resistance (mean +/- standard error of mean (sem)) of bacterial isolates derived from coral specimens collected at a site near and far (salmon and turquoise bars, respectively) from salmon farms in Comau Fjord, Chile. A summary of all isolates is shown on the left, and isolates are separated by colour type on the right. The numbers in brackets at the bottom of the bars represent the number of isolates per site and colour type. * denotes significance at p-values < 0.05.

Altogether 46 bacterial isolates of different colour were subjected to antibiotic resistance testing. The coral-associated bacterial isolates collected near the salmon farm had a significantly higher mean number of antibiotic resistances (7.7 ± 0.47 (mean ± s.e.m.)) than coral-associated bacterial isolates from the far site (6.3 ± 0.48, Fig. 1, aligned, rank-transformed ANOVA df = 1, F = 5.2818, *p* = 0.036). This difference was observed in all colour types, but was only significant for the rare types (df = 40, t = −3.48, *p* = 0.025).

### Molecular taxonomic identification of selected bacterial isolates

In general, coral homogenate samples obtained from the site far away from the salmon farms were characterized by more CFUs compared to samples from the near site (Fig. S1). The representative subset of bacterial isolates (in terms of colony characteristics) used for the antibiotic testing covered diverse phylogenetic classes: Gammaproteobacteria (37 isolates), Alphaproteobacteria (3 isolates), Bacilli (3 isolates), Actinomycetes (2 isolates) and Flavobacteriia (1 isolate). Thirteen genera were identified: *Pseudoalteromonas* (15 isolates), *Shewanella* (12 isolates), *Vibrio* (6 isolates), *Pseudovibrio* (3 isolates), *Microbacterium* (2 isolates), and 1 isolate each for the genera *Aliivibrio*,* Kordia*,* Pseudalkalibacillus*,* Cognaticolwellia*,* Stutzerimonas*, *Planococcus*, *Bacillus* and *Psychrobacter*. The site near to the salmon farm was characterized by higher numbers of *Shewanella* compared to the far site (8 versus 4 isolates), but lower numbers of *Pseudoalteromonas* (5 versus 10 isolates) and *Vibrio* (2 versus 4 isolates). Overall, the cultured microbiome indicated a shift from a *Shewanella*-dominated microbiome near the salmon farm to a *Pseudoalteromonas*-dominated microbiome far from the salmon farm (supplementary table S1,2).

### Antibiotic resistance profiling of selected bacterial strains

**Fig. 2:**
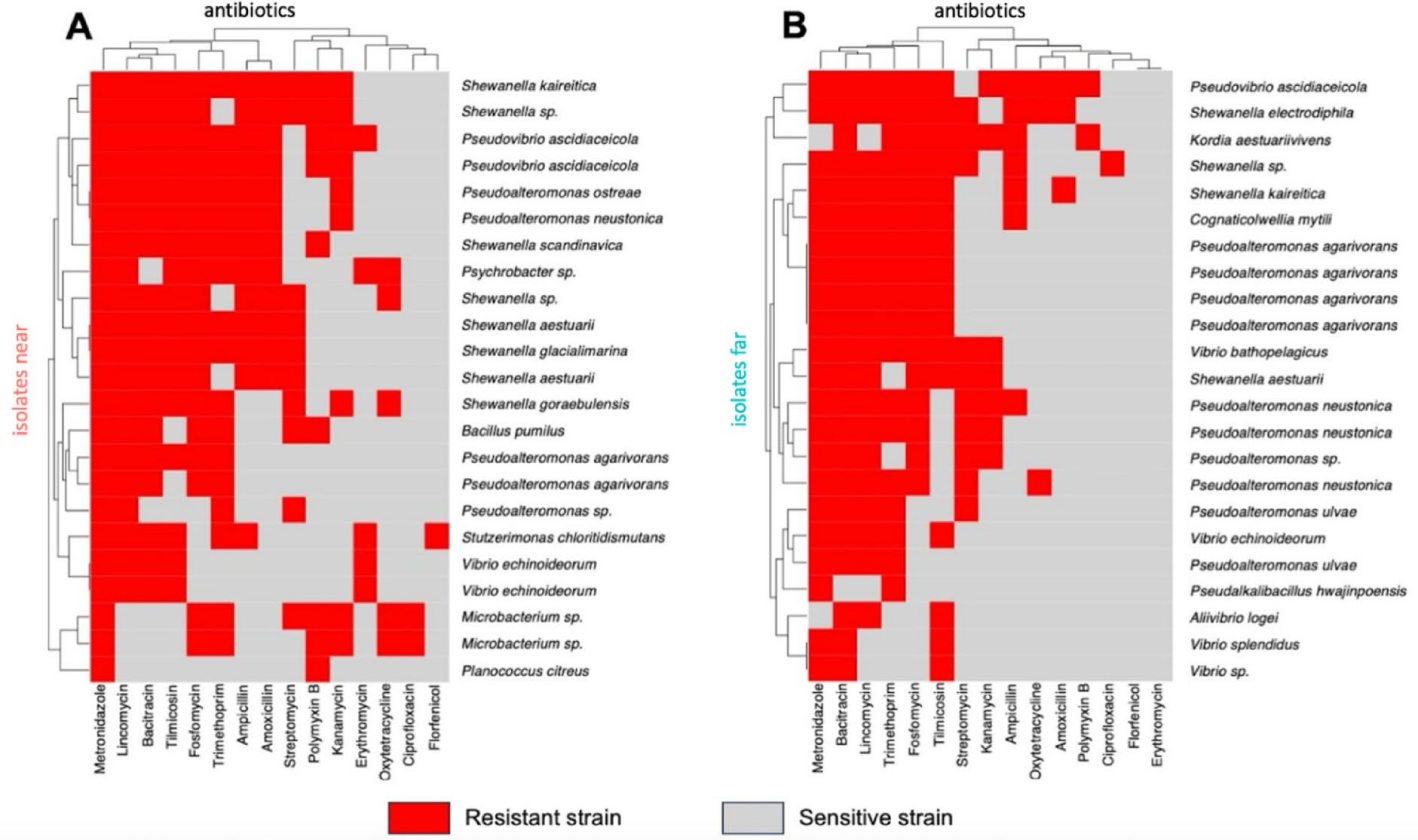
Resistances of selected coral-associated bacterial isolates to 15 antibiotics. Heatmaps showing the resistance of the selected *Desmophyllum dianthus*-associated isolates against 15 different antibiotics at the sites near (**A**) and far (**B**) from the salmon farms in Comau Fjord, Chile. Heatmaps are based on hierarchical clustering of antibiotic resistance across selected isolates and antibiotics. Red colour indicates isolate resistance and grey colour shows isolate sensitivity to the respective antibiotics.

All isolates displayed antibiotic resistance to at least 2 antibiotics and as many as 11 different antibiotics. At the near site, resistance was observed primarily against the antibiotics metronidazole (23 out of 23 isolates), bacitracin (18 out of 23 isolates) and lincomycin (20 out of 23 isolates), tilmicosin (17 out of 23 isolates), and fosfomycin (18 out of 23 isolates, Fig. 2). At the far site, resistance was observed most commonly against the antibiotics metronidazole (21 out of 23 isolates), bacitracin (22 out of 23 isolates), lincomycin (19 out of 23 isolates), trimethoprim (18 out of 23 isolates) and fosfomycin (16 out of 23 isolates). The isolates showed sensitivity primarily to the antibiotics florfenicol (1 out of 23 isolates), ciprofloxacin (2 out of 23 isolates), oxytetracycline (5 out of 23 isolates), erythromycin (5 out of 23 isolates), and kanamycin (9 out of 23 isolates) at the near site, compared to the antibiotics erythromycin (0 out of 23 isolates), florfenicol (0 out of 23 isolates), ciprofloxacin (1 out of 23 isolates), polymyxin B (2 out of 23 isolates), and amoxicillin (3 out of 23 isolates) at the far site. For a given taxon, the isolates exhibited largely similar response to the same antibiotics, and no strong site-specific difference could be observed. Interestingly, most isolates were sensitive to the antibiotics used in salmon farming (e.g., florfenicol, oxytetracycline, erythromycin), and this observation was independent from the collection site. A noticeable exception was tilmicosin, to which the majority of isolates were resistant.

Most isolates across most genera displayed multidrug resistances to ≥ 5 antibiotics (Fig. 2). This pattern was independent of the collection site. All *Shewanella* isolates displayed multidrug resistance against at least 7 to 11 different antibiotics. All *Pseudovibrio* strains were resistant to > 10 antibiotics. Most *Pseudoalteromonas* showed multidrug resistances against 5 to 9 different antibiotics, but *P. ulva* and *Pseudoalteromonas* sp. showed resistances against only 4 antibiotics. The *Vibrio* isolates displayed multidrug resistances against 3–8 different antibiotics and one related *Aliivibrio* isolate was resistant to 3 antibiotics. Isolates of the genera *Planococcus* and *Pseudoalkalibacillus* were largely sensitive with resistances against ≤ 2 antibiotics.

**Fig. 3:**
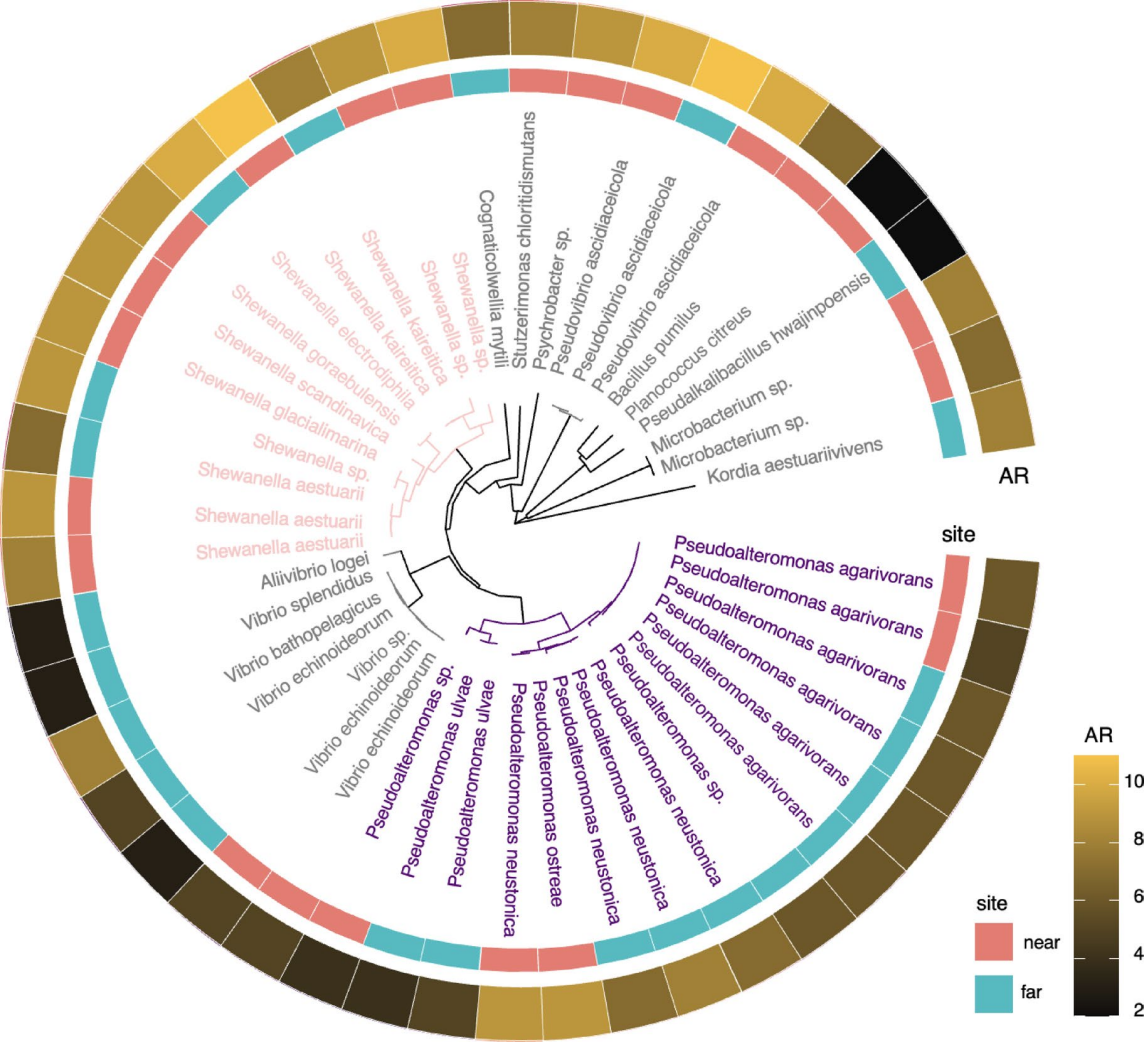
Bacteria associated with *Desmophyllum dianthus* based on 16 S rRNA gene sequences. Circular phylogenetic tree of coral-associated bacterial isolates at two sites near (salmon colour) and far away (turquoise colour) from salmon farms in Comau Fjord, Chile. Colour gradation from dark brown to yellow bars indicate the number of antibiotics to which the isolates are resistant (AR). The light salmon branch colour indicates the genus *Shewanella* and the purple colour indicates *Pseudoalteromonas*. The phylogenetic tree was constructed using the Neighbour-Joining model (Saitou and Nei 1987) in combination with the p-distance method. Bootstrap method was used to test phylogeny by running 1000 bootstrap replications.

The increase in antibiotic resistance at the near site was due to the presence of multiresistant isolates, such *Shewanella kaireitica* (11), *Pseudovibrio acidiaceicola* (11, 10), *Shewanella* sp. (10), and *Pseudoalteromonas ostrea* (9, Fig. 2A), and the dominance of *Shewanella* isolates in general (Fig. 3). In contrast to the far site, where the community was dominated by *Pseudoalteromonas* isolates (Fig. 3), yet the most resistant bacterial isolates belonged to *Pseudovibrio acidiaceicola* (11), *Shewanella electrodiphila* (10), *Shewanella* sp. (9), and *Kordia aestuariivivens* (8, Fig. 2B). On the other hand, only 2 isolates were resistant to ≤ 4 antibiotics at the near site compared to 5 bacterial isolates at the far site. These highly antibiotic-sensitive isolates at the far site include isolates that were not among the tested isolates obtained from corals near salmon farms such as *Aliivibrio logei*, *Vibrio* sp., *Vibrio splendidus* and *Pseudalkalibacillus hwajinpoensis* (Fig. 2).

### Antimicrobial activity of *D. dianthus*-associated isolates and antibiotics effects on culturable bacterial abundance

In general, only few antagonistic interactions were observed among the bacterial isolates tested, irrespective of testing within or between sites or between the genera *Pseudoalteromonas* and *Shewanella*. The only antagonistic activity was found by *Pseudoalteromonas agarivorans*, which slightly inhibited *Pseudoalteromonas ulvae* and *Pseudoalteromonas* sp. at both sites. Whether this activity is due to the agarolytic nature of this isolate or due to antimicrobial activity cannot be ascertained.

At the bacterial community level, bacterial growth was significantly higher from corals collected far from salmon farms (*p* = 0.019, Fig. S1A). Furthermore, the antibiotic supplements (both oxytetracycline and tilmicosin) in the growth media reduced bacterial growth in both sites to more or less the same level (no significant difference, Fig. S1B) and thus, growth inhibition was stronger at the far site compared to the near site (Fig. S1C).

### Analysis of microbial diversity and composition of *D. dianthus* microbiome

**Fig. 4:**
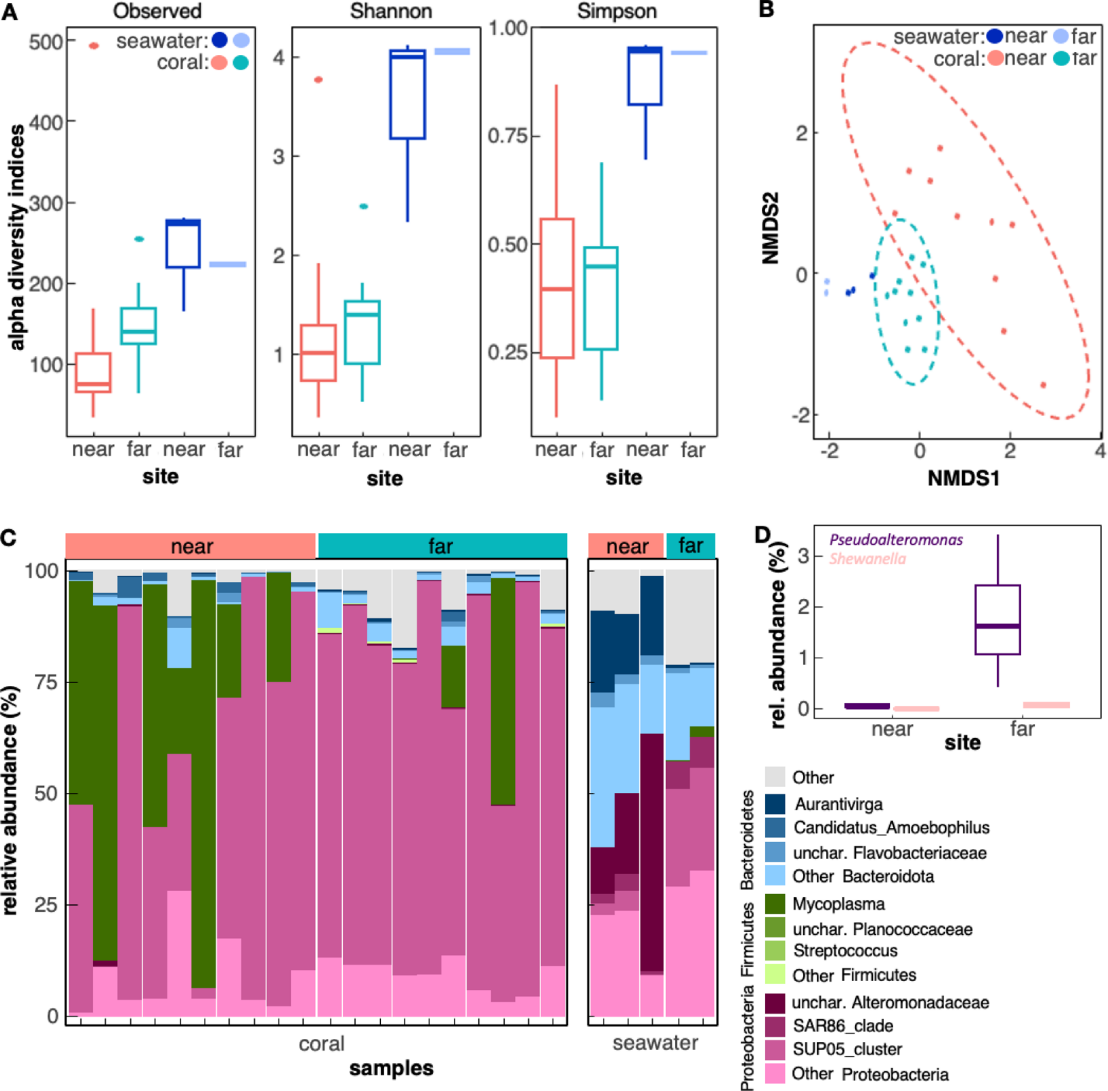
Microbial community characteristics in the cold-water coral *Desmophyllum dianthus* and seawater samples near and far from salmon farms in Comau Fjord, Chile. (**A**) Alpha and beta diversity assessment of the microbiome community. Alpha diversity indices (Observed, Shannon, and Simpson) are represented as boxplots for corals and seawater at the two sites. Corals are represented by the colours salmon and turquoise, and seawater by dark and light blue for sites near and far from salmon farms, respectively. (**B**) Dissimilarities in microbial community composition of corals and seawater samples from the two sites are represented by non-metric multidimensional scaling (NMDS) plots using Bray-Curtis distance metrics. (**C**) Prokaryotic community composition in coral and seawater samples at sites near and far from salmon farms. Stacked bar plots reveal proportional distribution in percentage of the three most dominant phyla (purple: Proteobacteria (Pseudomonata), green: Firmicutes (Bacillota), blue: Bacteroidetes (Bacteroidota)) and the three most dominant genera within phylum. All other taxa are grouped within others. (**D**) Relative abundances in percentage across sites are shown for the two genera *Pseudoalteromonas* (purple) and *Shewanella* (pink) that dominated the culture-dependent microbial communities and are grouped as others in C.

Metabarcoding analyses revealed significant differences in α-diversity indices (Observed, Shannon, Simpson) between corals and seawater, but showed little variation between sites (Fig. 4A). Corals were characterized by a reduced Shannon (diversity) as well as Simpson (evenness) index compared to seawater and thus, the dominance of a few taxa in the corals. Across both sites, bacterial diversity and evenness were very similar. Species richness (Observed) appeared lower at the near site, but this was not significantly different (Kruskal-Test, *p* = 0.191). The microbial community composition differed significantly among the seawater and coral groups (PERMANOVA: F = 12.118, R² = 0.63, *p* < 0.001; 9999 permutations, Fig. 4B). In addition, pairwise comparison revealed distinct communities were associated with corals from the two sites (Permanova near vs. far, p_adj_ = 0.021). This is also reflected at the level of the most common bacterial taxa, as the corals were dominated by *SUP05 cluster* and *Mycoplasma*, the latter being more common in corals near salmon farms (Fig. 4C). Different taxa were present in coral and seawater samples from both sites (Fig. 4C). The two dominant genera isolated from the coral tissue homogenate, *Pseudoalteromonas* and *Shewanella*, were present in the culture independent microbial community at all sites, albeit at low relative abundances (Fig. 4D). *Pseudoalteromonas* was found in higher relative abundance at the far site (1.8% ± 0.3), compared to the near site (0.09% ± 0.04), while the abundance of *Shewanella* was in general low, but slightly higher at the far site (0.07% ± 0.02 compared to near site 0.01% ± 0.005). However, *Pseudoalteromonas* was 34-times more common than *Shewanella* at the far site, compared to 2.9-times at the near site.

## Discussion

In this study, we combined cultivation-based with 16 S rRNA gene amplicon sequencing to assess the microbial associations of the CWC *D. dianthus* collected from two sites in Comau Fjord that differed in their proximity to salmon farms. In addition, we screened cultured bacterial isolates for antibiotic resistance capacity. Notably, the two sites showed distinct coral phenotypes, in line with previous work noting the stunted coral size with reduced tissue-covered surface area^22^ at the site near salmon farms. The visible differences were correlated with a higher fraction of *Pseudoalteromonas* isolated from corals at the far site compared to a *Shewanella*-dominance in the isolates at the site near the salmon farms. Similarly, we saw a change in microbial community composition based on amplicon sequencing between sites, shifting from a *SUP05 cluster*- to a *Mycoplasma*-dominated community, respectively. This change in dominance in bacterial isolates was accompanied by a slight but significant increase in antibiotic resistance close to salmon farms. While this only represents a pilot study with a limited number of isolates tested, the findings suggest that salmon farm activity with its antibiotic application may alter microbial associations in benthic organisms, like the cold-water coral *Desmophyllum dianthus*.

In our study, we observed an emerging pattern with distance to the salmon farms and coral health (shift in coral phenotype from larger in size with high tissue cover to smaller corals with reduced tissue cover^22^ and at the same time, the dominating bacterial taxa in the culturable coral-associated microbiome were *Pseudoalteromonas* at the far site compared to *Shewanella* at the site near the salmon farms. Both taxa, *Shewanella* and *Pseudoalteromonas*, have been described as members of the culturable coral microbiome in previous studies, in particular the latter. For example, P*seudoalteromonas* isolates contributed to approx. ⅓ of a total of 200 isolates from the CWC *Desmophyllum pertusum* at several sites within the Gulf of Mexico^28^ and showed high antibacterial activity against coral pathogens, in particular against the coral pathogens *Vibrio coralliilyticus* and *Thalassomonas loyana*^29,30^. Most *Pseudoalteromonas* are presumed to play an important role in host defence against pathogens^29,31^. They were preferentially isolated from coral mucus^32^ that suggest a more direct interaction with the surrounding seawater and higher susceptibility to changes in the environment. It is conceivable that the *Pseudoalteromonas* isolates obtained in the present study might perform similar functions in the *D. dianthus* microbiome, ranging from bacterial/pathogen defence and fouling prevention to nutrient acquisition from coral mucus. Yet with the observed lower antibiotic resistance compared to the other dominant taxa, they may be more susceptible to antibiotic release in their surrounding environment and thus, the first to be affected. There are two lines of evidence in support of this assumption: First, cultured bacteria experienced stronger losses when marine agar plates were supplemented with antibiotics in the *Pseudoalteromonas*-dominated coral tissue homogenates from the site far from salmon farm activity, compared to the *Shewanella*-dominated near site. Second, the culture-independent bacterial composition showed an 18-fold lower relative abundance of *Pseudoalteromonas* at the site near the salmon farms.

The role of the other dominant taxon, *Shewanella*, is less clear. While it was found in the cultured microbiome of corals^28,33^, it was less prevalent and abundant. The genus is considered psychrophilic, i.e., adapted to cold and extreme deep-sea environments^34^, which is why its presence in the microbiome of a CWC is not too unexpected. It potentially contributes to well-being of the CWC^33^ and could therefore form an integral part of the CWC holobiont’s microbiome even outside its usual habitat. *Shewanella* is expected to play an important role in energy and nitrogen cycling^35–37^. *Shewanella* strains were also found to be associated with farmed Atlantic salmon, specifically the gill tissue, and predominantly observed to be associated with healthy gill tissue^38^. The increased occurrence of *Shewanella* bacteria in farmed fish, however, may result from the enrichment through by-products, food pellets, and salmon excretions^39,40^. A similar enrichment may explain the increased presence in the coral culture-dependent microbiome near salmon farms in our study. *Shewanella* strains increased in abundance in tropical corals exposed to stressful conditions and potentially served as the first sign of stress^41^. Yet, it is not entirely clear whether their role is damaging or rather protective, as they may contribute to the degradation of metabolites produced as a response to stress^41,42^. Some *Shewanella* are clearly pathogens for marine animals^43^, and in the last two decades, *Shewanella* has attracted attention in relation to its antibiotic resistance and as progenitors of antibiotic resistance genes of clinical concern^44^. Given its ambiguity, we cannot conclude if *Shewanella* has a positive, negative or neutral effect on *D. dianthus*. While we do see a shift in cultured-microbial isolates that possibly is related to salmon farm activity, it is based on a limited number of bacterial isolates that warrant further assessment. With *Pseudoaltermonas*, we clearly find a dominant genus that we would expect for the cultivated microbiome of CWC, but the shift towards *Shewanella* is less clear, and its role in the coral holobiont should be investigated in more detail.

The most robust evidence for site-specific differences emerged from amplicon sequencing, which revealed a clear shift in dominant taxa from the SUP05 cluster far from salmon farms to *Mycoplasma* dominance near salmon farms. This may provide an additional indication that the prevalent conditions at the two locations favour different microbial taxa. The SUP05 cluster was found to increase in relative abundances in coral hosts near active cold seeps^25^ and to contribute to nitrogen and carbon fixation as well as sulphur oxidation. It was also suggested to provide essential amino acids, nitrogen, or vitamins for the cnidarian *Paramuricea* sp. in the Gulf of Mexico^45^. Similarly, the detection of *Mycoplasma* as a member of the CWC-associated bacterial community is becoming more widespread^46,47^, but the role of *Mycoplasma* in corals is still debated. As a common and sometimes even dominating taxon in the microbiome of especially cold-water octocorals, *Mycoplasma* was suggested to be a commensal or mutualist for the corals^46,48,49^. By contrast, some members of this genus have been described as human and animal pathogens^50^. Our findings suggest a more opportunistic than mutualistic role, given that *Mycoplasma* became dominant only in the stunted CWCs near the salmon farm. An evaluation that is also in agreement with those of Palladino et al.^51^ with a higher relative abundance of the family Mycoplasmataceae (9.4 ± 16.5% in samples close to aquaculture vs. 0.8 ± 3.3% in controls) in the limpet *Patella caerulea* near an aquaculture site in southern Sicily. Thus, it may be possible that the higher relative abundance of *Mycoplasma* is related to some degree to salmon farm activity. The bacterial group of *Mycoplasma* is certainly of great interest within CWC holobionts, in particular due to its high dominance in this region in some *D. dianthus* specimens, and definitively deserves more detailed investigations regarding its actual role and functionality for CWCs.

**Fig. 5:**
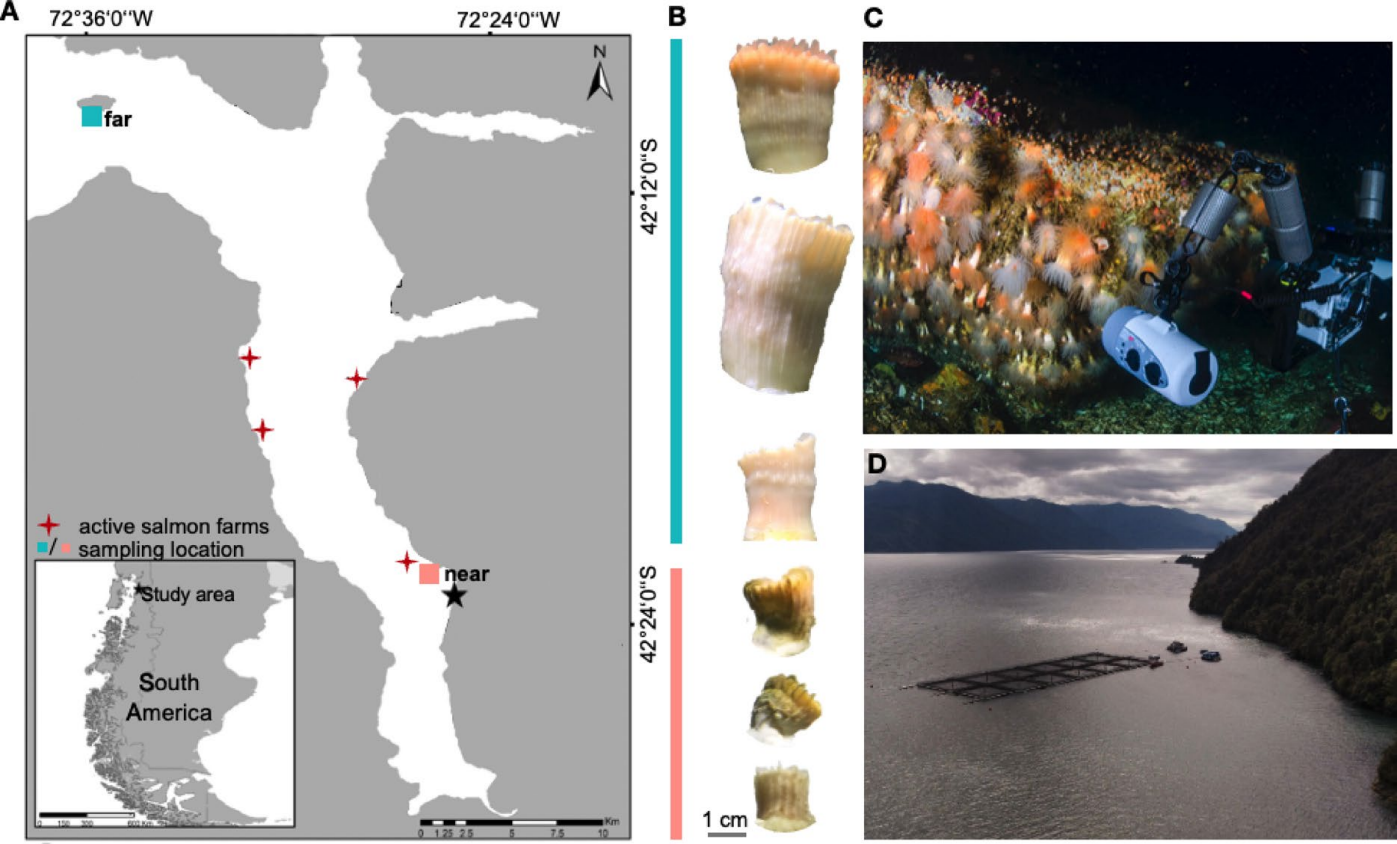
Study area in Comau Fjord in Patagonian Chile. (**A**) Map of the study area. The study sites were chosen near (salmon colour) and far (turquoise) from salmon farm activity. Salmon farm locations (active salmon farms at the time of coral sampling) in Comau Fjord are indicated by red crosses. (**B**) Representative individuals of the cold-water coral *Desmophyllum dianthus* collected far and near to salmon farm activity. (**C**) *Desmophylum dianthus* can form dense coral banks in this fjord in shallow waters (photo credit Thomas Heran Arce). (**D**) However, this region is also known for its intense aquaculture, in particular salmon farming (photo credit U. Pörschmann).

An important background to this work is that the two locations differed in terms of the expressed coral phenotype, with a suppressed phenotype observed in the vicinity of the salmon farm. This suppressed phenotype can certainly contribute to a better understanding of the response of CWC holobionts to limiting environmental conditions^22^. To date our understanding of CWC health is sparse and more data are required. The few studies with clearly distinct tissue health conditions similarly found shifts in dominating microbial taxa, especially when the tissue becomes diseased or necrotic compared to healthy looking tissue in CWCs^26,27^. CWC physiological studies are still lacking that assess coral performance traits alongside the prevalent environmental conditions the corals thrive in to better integrate such data. CWC performance may already be compromised before the tissue appears unhealthy or necrotic. Here we find healthy looking tissue but compromised performance (stunted size, reduced tissue cover, cf., Fig. 5^22^, along with changes in CWCs’ microbial associations shifting towards *Shewanella* or *Mycoplasma*. Interestingly, Hornick and Buschmann^52^ also showed an enrichment of *Shewanella* together with a decrease in bacterial diversity in sediments near a salmon farm in the Chiloé area, Chile. Since most microbiome studies near aquacultures have so far focused on seawater and sediments^17,52,53^, this is one of the first studies in the region on a benthic organisms and the observed change in dominant bacterial taxa may possibly result from aquaculture activity. Regardless, our findings can serve as an early indicator of environmental changes affecting CWC holobionts, even when coral tissue appears visually healthy.

It is noteworthy that we observed differences in the bacterial species isolated and cultured from the coral specimens in comparison to amplicon sequencing of coral holobionts, which is not unusual and is a known phenomenon (aka “great plate count anomaly”^54^. The composition of the culturable *D. dianthus*-associated microbiome, however, is overall in agreement with previous coral studies. Genera like *Pseudoalteromonas*,* Vibrio*, and *Pseudovibrio* are frequently isolated from corals^28,32^ and so are the genera *Psychrobacter*, *Planococcus*, *Bacillus* isolated in this study, albeit in lesser numbers in previous studies. Similarly, bacterial isolates from the closely related CWC *Desmophyllum pertusum* were dominated by *Pseudoalteromonas* at several sites in the Gulf of Mexico (approx. ⅓ of a total of 200 isolates). In addition, *Shewanella* have also been isolated from this species^28^. While in the metabarcoding assessment different microbial taxa dominate the communities, the assessment aligns in one way with culturable microbial isolate assessment as it confirms the reduced bacterial richness as well as stronger loss of antibiotic-sensitive isolates like *Pseudoalteromonas*. Together, this may indicate a restructuring role of antibiotics released into the environment on the microbiome of benthic organisms. A similar restructuring effect by aquaculture activity was observed for the diversity of benthic macroinvertebrates in general^51,55^ as well as their associated microbes^56^.

The differences in the culturable microbial communities between the sites far and near from salmon farms were accompanied by high antibiotic resistance profiles. A generally high resistance (42–51% of tested antibiotics) was observed across sites with a slightly but significantly higher resistance in bacterial isolates from corals collected near salmon farms (Fig. 5). For some bacterial isolates, a specifically high resistance (11 antibiotics or 73%) was detected (Figs. 2 and 3). Antimicrobial resistance in natural environmental settings is not unusual, as it is part of a bacterial defence and survival strategy^57,58^. Even in putatively pristine habitats, like Arctic fjord water and sediments, bacterial strains have been found with high antibiotic resistance^59^. Yet anthropogenic activity, like eutrophication and pharmaceutical waste, can alter antibiotic resistance in natural microbial populations^57,58^. To put the identified antimicrobial resistance profiles into context, baseline data are needed to understand natural resistance values and link observed changes to potential anthropogenic drivers. However, in most cases such baseline data are lacking^60^, let al.one the values for antibiotic resistance in CWC-associated bacteria. The host uses a number of mechanisms, including antimicrobial peptides, to shape its resident bacterial community^61^. The host-associated microbiota, hence, need to be able to deal with antimicrobials, which may have contributed to the rather high antimicrobial resistance values in our study.

The only study so far that tested for antibiotic resistance in CWC-associated bacteria, found a minimum resistance of 17% (1 out of 6 antibiotics) of all antibiotics tested, a maximum of 67% (4 out ot 6), and an average of 43.2 ± 9.9% (2.6 ± 0.6 antibiotics out of 6) across the bacteria isolated from *D. pertusum*^28^. Even though this study is not directly comparable to ours (different numbers and antibiotics tested), the relative antibiotic resistance values are similar to values obtained from the far site (42 ± 3.2%), yet slightly lower compared to the site near salmon farms (51.3 ± 3.1%). While this confirms that antibiotic resistance can be high in natural environments, it may also suggest anthropogenic effects on antibiotic resistance levels. Changes in resistance were found most prominently in freshwater systems, where the release of antibiotics affected not only bacterial resistance but also bacterial diversity of downstream habitats^62^. Similarly, mariculture in coastal habitats leads to the introduction and spread of antibiotic resistance genes (ARGs)^63,64^. In our study, *Shewanella* was characterized by high multi-resistance values of 9 ± 0.3 compared to 6.27 ± 0.4 in *Pseudoalteromonas* across sites. Within *Shewanella*, even the same isolates can differ in antibiotic resistance, which was observed in *S. aestuarii* and *S. kaireitica* from near vs. far sites in the present study (with resistance of 8.5 vs. 7 and 11 vs. 8 antibiotics, respectively). A similar enhancement was found in *S. aestuarii* isolated from a Portuguese estuarine habitat, where one isolate was susceptible to all antibiotics tested, whereas the other one was only resistant to four out of 11^44^. Therefore, the patterns of antibiotic susceptibility are not always associated with the respective bacterial strain, suggesting the presence of other mechanisms like gene transfer and mobile genetic elements. Even in *Pseudoalteromonas* we find such an increase in antibiotic resistance (*P. neustonica*), though not in all isolates (e.g., *P. agarivorans*). It has been recognized that certain marine bacterial taxa (e.g., *Vibrio*, *Shewanella*) are reservoirs as well as vehicles of antibiotic resistance^44,63,64^ through the exchange of mobile genetic elements even across taxa^65^. Such mechanisms may be responsible for the observed increase in antibiotic resistance in the same bacterial taxa isolated in this study across or even within sites (*S. aestuarii* and *S. kaireitica*). A number of ARGs were detected in seawater bacteria of Comau Fjord and it was shown that they conveyed resistances to beta-lactams, tetracyclines, bacitracin and the group macrolide–lincosamide–streptogramin^17^. A recent study of antibiotic-resistant bacteria and genes in seawater off Chiloé Island showed that this can result in significant spatial differences with an increase near salmon farms^53^.

It should be borne in mind that our preliminary observations on CWCs in the context of aquaculture do not rule out the possibility that several other factors may have influenced the observed microbial community and AMR patterns. For instance, local confounding factors are the vicinity of a small settlement affecting the near site, the breeding colonies of seabirds and sealions affecting the far site, or the oceanographic differences between a protected semi-enclosed fjord (near site) and exposed mouth of the fjord (far site). On the other hand, advection and mixing may have contributed to homogenizing the water masses, thus dampening the differences between near and far sites^66^. This was seen in previous studies that did not find large differences between sites separated by up to 8 km^16,67^. Dispersal of antibiotics by advection is suggested by the traces of florfenicol in a distant control site^16^ as well as the expected general long persistence times of such chemicals in the environment^68^. Given that the aquaculture activity of salmon farms changes between years, antibiotic persistence may induce delayed effects. Despite the overall increase in salmon aquaculture, we found low resistance of bacteria to the most commonly used antibiotics in Chilean salmon farming in the present study (Fig. 2). While this is to some extent unexpected, it aligns with previous studies that also found a rather low resistance to the most common salmon mariculture antibiotic, florfenicol^16,69^. In fact, this antibiotic is widely used in agriculture and aquaculture because it has proven effective against bacterial infections. Its strong effect on a range of both Gram-positive and Gram-negative bacteria has raised concern that it may alter microbial communities in the natural environment^70^ and neighbouring habitats.

## Conclusion

We here report preliminary observations from the microbiome of the CWC *D. dianthus* collected at two sites in Comau Fjord, Chile, near and far from the influence of salmon aquaculture. The CWCs differed both in their phenotypic characteristics and microbiome between sites. The culturable fraction of the associated microbiome was dominated by *Pseudoalteromonas* at the far site compared to a *Shewanella*-dominated fraction of the microbiome at the site near the salmon farms. This difference was accompanied by a slight but significant increase in antibiotic resistance of coral-associated bacteria near salmon farms. Similarly, we saw a clear change in microbial community composition based on amplicon sequencing between sites, changing from a *SUP05 cluster*- to a *Mycoplasma*-dominated community near salmon farms. Our findings add to a growing body of independent studies suggesting that salmon farms may change microbial associations and be a source of increased antibiotic resistance in natural ecosystems. They expand the scope from seawater and sediments to the little-known benthic species. They fuel concerns that salmon farming may affect the microbiome and metabolic performance of corals, which are key foundation species in Patagonian fjords. Future studies are needed to validate the preliminary findings. They should include replicate sites within and across fjords and combine the distribution of antibiotics in the environment with a metagenomic assessment of the microbial community to screen for changes in ARGs.

## Materials and methods

### Coral collection site and coral processing

The Comau Fjord in the northern part of Chilean Patagonia (42°10′ S; 72°40′ W) is part of the Chilean fjords system (Fig. 5A). The Fjord is 41 km long, 4.5 km wide and up to 480 m deep and connects with the open ocean through the shallow Gulf of Ancud. It is known for its dense and widely distributed populations of CWCs found across the entire fjord from head to mouth^20^ and is one of the few areas in the world featuring deep-sea emergence, i.e., the occurrence of CWCs near the surface^71^. *Desmophyllum dianthus* specimens for this study (Fig. 5D) were sampled in Comau Fjord by focusing on two sites that have been established in a previous study^22^ namely Station C (42°22’ S 72°25’ W) and F (42°09’ S 72°35’ W, Fig. 5). These two sites were ideal for this study as one site is located right next to a salmon farm (sampled on the wall 0.4 km away from the farm shown in Fig. 5D), while the other site is located far away from the next active farm (18–20 km). In addition, three salmon farms in the centre of the fjord are also located closer to the sample site right next to an active salmon farm (12–15 km), which is expected to affect the site near the salmon farm more than the far site. The corals were sampled in October 2021 and prepared for in-situ transplantation work (Wall et al. in preparation). Information on the activity of salmon farms in the region was gathered from the Chilean National Fisheries and Aquaculture Service (Servicio Nacional de Pesca y Acuicultura - SERNAPESCA) and active farms are indicated in Fig. 5A.

Corals were chiseled from the fjord walls by scientific SCUBA divers at approx. 25 m depth. The corals were placed in 1 l Kautex bottles while underwater and transported to the laboratory in a cooler filled with ambient seawater. Back in the laboratory, the corals were maintained in a flow-through aquarium system with water pumped from 20 m depth and prepared for returning to the field following established procedures^22^. Briefly, corals were cut and glued onto labelled plastic screws. One year later, corals were recollected and weighed before processing the samples for microbial analyses for both culture-dependent and culture-independent techniques of the present study. For subsampling of the corals, they were gently rinsed with 0.2 μm filtered seawater to remove loosely associated particles and bacteria. Each coral individual was sub-divided into pieces of approx. ⅛ of the individual and these were then either placed into sterile 2 ml cryovials and immediately snap-frozen in liquid nitrogen for amplicon sequencing or they were supplemented with 5 ml of 20% (v/v) glycerol in sterile seawater and sterile 2 mm glass beads for bacterial cultivation^72^. The latter tubes were then vortexed for 2 min to produce coral-associated bacterial homogenates. The glycerol cell suspension was transferred into two sterile Eppendorf tubes and stored at −80 °C until further processing. Note that our procedure (chiseling, mounting on screws, transplantation and resampling) followed established procedures in coral research, including microbiome assessments^73,74^. While we cannot fully rule out that these manipulations may have led to unforeseen changes in the microbiome, we can rule out handling bias, e.g. differences in manipulation between the two sites that would have led to the observed differences in the microbiome, as all corals underwent the same handling procedure. In addition to the corals, up to three seawater samples of 1–2 l each per collection site were taken with a Niskin bottle next to the coral site for assessment of the seawater microbial community. Seawater samples were filtered through a 0.2 μm bacterial filter. Filters were placed in sterile 2 ml cryotubes and immediately snap-frozen in liquid nitrogen.

### Cultivation and preservation of coral-associated bacteria

Bacterial cultivation from *D. dianthus* coral tissue homogenates was performed on Marine Agar, consisting of 37.4 g BD DifcoTM Marine Broth 2216 (Becton Dickinson and Company, USA), 18 g Bacto-Agar (Otto Nordwald GmbH, Germany), 1000 ml deionized water, and adjusted to pH 7.7. Bacterial growth was first tested at two different temperatures (+ 12º C, + 20º C) and three dilutions of each homogenate (undiluted sample, 10 − 1, 10 − 2 dilutions). Serial dilution of coral homogenate was performed using saline solution (32 g/l sea salt, Tropic Marine^®^), and homogenates were spread onto marine agar plates in duplicates. A higher number of bacteria in far-site samples required dilution (10^− 1^) for the final cultivation procedure to be able to isolate individual colony-forming units (CFUs), but not for corals collected at the near site. Based on these preliminary tests, + 20 °C was identified as optimal for culturing coral-associated bacteria as the CFU counts were higher and the diversity of colony morphotypes was unaffected. Most coral-associated bacteria were thus isolated at + 20º C within 3–7 days. Colonies were selected randomly but representative for the overall diversity, covering many different colony morphotypes. Colonies were purified by repeated streaking until visually pure. The Cryobank System (Mast Diagnostica GmbH, Germany) was used to deposit 3-day-old agar plate cultures in duplicate. This system allows long-term preservation at −80º C on chemically treated beads with cryogenic preservative solution. A total of 88 strains obtained from 14 coral individuals were deposited and maintained at the GEOMAR Biobank of the Marine Symbiosis Research Unit.

### Antibiotic resistance testing of bacterial isolates

Bacterial colonies were initially grouped based on their colony characteristics. Most colony morphotypes belonged to the ‘white type’ (white, milky, and beige coloured colonies), to a slightly lesser extent to the ‘orange type’ (orange, peach, and yellow colonies), and only a few to the ‘rare colour type’ (translucent, black, or light-yellow colonies). This selection for colours is based on literature observations stating that pigmentation can be an indicator of antibiotic resistance or, more generally, of antagonistic interactions^75^. To not bias the antibiotic resistance testing, we selected a subset of 23 bacterial strains from the near and far site (46 strains in total), selecting colony colour types that were representative for the overall culturable bacterial community. Altogether 11 ‘white’, 9 ‘orange’, and 3 ‘rare’ colour types were chosen for each site that were representative of the cultured bacterial diversity (in terms of colony characteristics) and represented seven coral individuals per site. These isolates were screened against 15 different antibiotics (Oxoid Company) using the Kirby-Bauer disc diffusion method^76^. Concentrations were chosen according to the literature^16,67^ or following common use in medical practice (from 5 µg to 50 µg and from 10 IE to 300 IE). The antibiotics were selected based on their use in the Chilean salmon farming industry and in general human medicine, and together they cover all major classes of antibiotics (supplementary table S3). It is important to note that, according to SERNAPESCA (pers. communication), the first four antibiotics (florfenicol, oxytetracycline, tilmicosin, and erythromycin) are commonly used in salmon farming in the Chilean fjord system.

Antimicrobial activity was determined on 3-day-old cultures. Two to three antibiotic discs were placed on agar plates and monitored for inhibition zones after 3 days of cultivation at 20 °C. Each strain was tested for each antibiotic at least twice, and in case the results were equivocal, tested a third time. The two-fold absence of an inhibition zone on day 3 was considered as strain resistance. The data were noted as yes or no answers, rather than focusing on nuanced differences in the diameter and/or clarity of the halos. Antibiotic resistance was considered present only if no zone of inhibition was observed.

### Antagonistic activity screening

The antagonistic activity of fresh one-day-old cultures of *Pseudoalteromonas* and *Shewanella* was tested by cross-streaking on 55% Marine Agar at room temperature. Diluted marine agar was used to compensate for the fast-growing nature of the bacteria and to improve the detection of potential inhibition zones. The antagonistic activity of the strains was assessed after 7 days between and within strains of each site, near and far from the salmon farms (*Pseudoalteromonas* vs. *Pseudoalteromonas* isolates, *Shewanella* vs. *Shewanella* isolates, *Pseudoalteromonas* vs. *Shewanella* isolates).

### Antibiotic resistance testing of bacterial communities from coral tissue homogenates

Serial dilutions of 7 coral homogenates were performed from each site near and far from salmon farms. Each homogenate dilution (undiluted sample and 10^− 1^ dilution) was plated in triplicate on three types of culture media (marine agar, marine agar with 75 mg/l tilmicosin (Sigma), and marine agar with 100 mg/l oxytetracycline (Sigma)). The bacteria were cultivated at + 20 °C for one month. The number of CFUs per plate was counted on days 3, 7, and 35 of the culture. All plates showing excessive bacterial growth (overgrowth of CFUs) were excluded from statistical analyses.

### Bacterial strain 16 S rRNA gene amplification and phylogenetic tree construction

Genomic DNA was extracted from 3-day-old cultures using the DNeasy Blood & Tissue Kit (Qiagen GmbH, Germany). Amplification of 16 S rRNA gene sequences was performed with primers Eub27F (5′-GAG TTT GAT CCT GGC TCA G-3′) (Sun et al. 2012) and Univ1492R (5′-GGT TAC CTT GTT ACG ACT T-3′)^77^. The amplification temperature regime entailed: 1 cycle of denaturation at 93 °C (2 min); 30 cycles of amplification at 55 °C (30 s), 72 °C (30 s), and 92 °C (30 s); 1 cycle of last elongation at 42 °C (1 min), 72 °C (5 min), and cooling at 10 °C. Primers 534R^78^, 342 F^79^, and Univ1492R^77^ were used for Sanger sequencing at Eurofins Genomics (Köln, Germany).

The 16 S rRNA gene sequence quality and contig assembly was assessed with ChromasPro 2.1.8 (Technelysium Pty Ltd, Australia). 16 S rRNA gene sequences were then compared with sequences from the NCBI database^80^ using the tool BLAST^81^. Sequence alignment and construction of the phylogenetic trees were performed in MEGA version 11.0.13^82^. The MUSCLE tool (UPGMA method) was used to align the sequences. Phylogenetic trees were constructed using the Neighbour-Joining model^83^ in combination with the p-distance method. Bootstrap method was used to test phylogeny by running 1000 bootstrap replications (NCBI GenBank PV088670-PV088715).

### Diversity of the *D. dianthus* microbiome assessed by amplicon sequencing

Small coral fragments of approx. 500 µl volume (n = 10 per site) and half a filter of seawater sample (n = 3) were used for the extraction of genomic DNA using the ZymoBIOMICS miniprep DNA/RNA kit (ZymoResearch, Irvine, CA) following the manufacturer’s instructions. Alongside the extraction, negative pipeline controls were run every second batch of extractions. The V5-V6 variable region of the 16S rRNA gene was amplified to obtain libraries of amplicons from all samples and additionally from three negative PCR control as well as two positive Mock community controls per well plate (ZymoBIOMICS microbial community DNA standard). The temperature regime for 16S rRNA gene amplification was as follows: 29 cycles at 98 °C (30 sec), 98 °C (10 sec), 52 °C (30 sec); 1 cycle at 72 °C (30 sec) and a hold at 4°C. 16S libraries were generated according to a dual-indexing amplification protocol^84^ using the primers 784 F (5’ -AGGATTAGATACCCTGGTA-3’) and 1061R (5’- CRRCACGAGCTGACGAC-3’), enabling individually tagging and further assigning of the sequence data. Sequencing was performed on a MiSeq platform (using the MiSeq Reagent kit v3, 600 cycles, PE300) at the Alfred Wegener Institute, Bremerhaven, Germany.

The raw sequences were demultiplexed, de-noised and ASVs (amplicon sequence variants) were generated after 16 S rRNA amplicon sequencing using QIIME2 v2020.11^85^ and DADA2^86^. Eukaryota, chloroplasts, mitochondria and Archaea were removed from the ASVs dataset. The dataset was checked for contamination post-sequencing with the *microDecon* package^87^ by using both negative and positive control (whole pipeline negative controls aka kit blanks, PCR controls and a Zymo Mock community as positive control) with threshold 0.7 to remove contamination from the 16 S rRNA amplicon data. The microbial community data after decontamination contained a total of 789.912 reads and 25.679 ASVs across samples, including rare ASVs. Sequences (deposited in NCBI GenBank PX715967 - PX716645) were analysed in R version 4.3.1 using *phyloseq*^88^ and *vegan*^89^ packages. The α-diversity and β-diversity of the coral-associated microbiota was assessed on the rarefied (size-normalized) dataset using the “rarefy-to-even-depth” function of the package *phyloseq.* The rarefied data set contained 2029 ASVs with an average of 6206 reads per sample. The α-diversity indices (Observed, Shannon, and Simpson) were analysed. The data were assessed for normality by the Shapiro-Wilk normality test and PERMANOVA analysis was performed for statistical analysis of culture-independent dataset.

### Data analysis

Data processing and visualization were conducted in R version 4.3.1 (R Core Team 2023) using, for instance, the packages *ggplot2*^90^. The heatmaps were constructed with the *ComplexHeatmap* package^91^ using default values for hierarchical clustering (euclidean distance and complete) of both bacterial isolates (rows) and antibiotic resistance (columns) values. Statistical differences in antibiotic resistance across sites and colour type (‘white’, ‘orange’ and ‘rare’) were assessed following a two-way full-factorial nonparametric procedure due to non-normal data distribution and unbalanced design. We used aligned, rank-transformed, non-parametric factorial analyses of Variance (ARTool ANOVA package^92^ with site and colour type as fixed factors (including interactions) and the coral genotype from which the isolates originated as a random effect.

## Supplementary Information

Below is the link to the electronic supplementary material.

Supplementary Material 1

Supplementary Material 2

Supplementary Material 3

Supplementary Material 4

Supplementary Material 5

## Data Availability

The datasets derived and analyzed for this study are available as supplementary material for the antibiotic resistance testing (Dataset 1 – strain specific antibiotic resistance findings, Dataset 2 – bacterial growth inhibition by antibiotics) and the countable for the 16 S rRNA (Dataset 3 - Overview over sequence counts and taxonomic classification per coral specimen). Sequencing data are deposited in the NCBI GenBank under the accession numbers (PV088670-PV088715, PX715967 - PX716645) also provided in the material and methods section for the respective datasets.
