# Supplementary material for: Changes in bacterial community composition and antibiotic resistance profiles of coral-associated microbiota in the vicinity of Chilean salmon farms
Source: Sci Rep. 2026 Jun 20;16:19185. doi: 10.1038/s41598-026-57517-y (PMC13283219; doi:10.1038/s41598-026-57517-y)
Supplement: Supplementary file 5 — Supplementary Material 5 [file 41598_2026_57517_MOESM5_ESM.docx]

## Supplementary material to

**Changes in bacterial community composition and antibiotic resistance profiles of coral-associated microbiota in the vicinity of Chilean salmon farms**

Anna Berezkina^1*^, Tanja Rahn^2^, Mailin Suchantke^2^, Kristina K. Beck^1,3^, Juan Pablo Espinoza^1,4,5^, Claudio Richter^1,5^, Ute Hentschel^2^ & Marlene Wall^1,2*^

**Supplementary Table S1.** Identification of the 23 selected bacterial isolates derived from the cold-water coral specimens from the near site.

| **Strain ID** | **Colour type** | **Closest relative strain by BLAST (Accession number)** | **Sequence length (bp)** | **Sequence Identity (%)** |
| --- | --- | --- | --- | --- |
| AB-84 | white | *Bacillus pumilus* strain NCTC10337 (LT906438.1) | 1488 | 99.80 |
| AB-78 | white | *Planococcus citreus* strain JCM 2532 ([LC379145.1](https://www.ncbi.nlm.nih.gov/nucleotide/LC379145.1?report=genbank&log$=nucltop&blast_rank=3&RID=WJ75AGP5016)) | 1490 | 99.80 |
| AB-98 | white | *Pseudoalteromonas agarivorans* DSM 14585 chromosome I, complete sequence  (CP011011.1) | 1478 | 99.73 |
| AB-113 | white | *Pseudoalteromonas agarivorans* DSM 14585 chromosome I, complete sequence (CP011011.1) | 1481 | 99.66 |
| AB-103 | white | *Pseudoalteromonas ostreae* strain hOe-66 (OQ472584.1) | 1485 | 99.46 |
| AB-82 | white | *Pseudoalteromonas neustonica* strain PAMC 28425 (NR_151996.1) | 1483 | 99.22 |
| AB-83* | white | *Pseudoalteromonas tunicata* strain D2 chromosome 1, complete sequence (CP031961.1) | 1484 | 98.52 |
| AB-76* | white | *Psychrobacter frigidicola* strain DSM 12411 (NR_042222.1) | 1481 | 97.84 |
| AB-79* | white | *Shewanella woodyi* ATCC 51908 chromosome, complete genome (CP000961.1) | 1490 | 98.26 |
| AB-77 | white | *Vibrio echinoideorum* strain DSM107264, (AP025483.1) | 1504 | 99.80 |
| AB-81 | white | *Vibrio echinoideorum* strain DSM107264, (AP025483.1) | 1500 | 99.87 |
| AB-132***** | **orange** | *Microbacterium caowuchunii* strain ST-M6 chromosome, complete genome (CP044231.1) | 1499 | 97.80 |
| AB-119* | **orange** | *Microbacterium lacus* strain A5E-52 (NR_041563.1) | 1464 | 98.07 |
| AB-112 | **orange** | *Pseudovibrio ascidiaceicola* strain NBRC 100514 (NR_113916.1) | 1427 | 99.65 |
| AB-85 | **orange** | *Shewanella aestuarii* strain JCM17801 (MT760375.1) | 1492 | 99.93 |
| AB-95 | **orange** | *Shewanella aestuarii* strain JCM17801 (MT760375.1) | 1487 | 100 |
| AB-89 | **orange** | *Shewanella goraebulensis* strain DAU312 (CP126972.1) | 1486 | 98.98 |
| AB-96 | **orange** | *Shewanella glacialimarina* strain TZS-4 chromosome, complete genome (CP041216.1) | 1492 | 98.80 |
| AB-91* | **orange** | *Shewanella kaireitica* strain c931 (NR_040951.1) | 1467 | 98.25 |
| AB-114 | **orange** | *Shewanella scandinavica* strain SP251-2 (NR_197676.1) | 1488 | 99.64 |
| AB-111 | rare | *Pseudovibrio ascidiaceicola* strain F423 (NR_041040.1) | 1427 | 99.71 |
| AB-110 | rare | *Shewanella kaireitica* strain c931 (NR_040951.1) | 1483 | 98.92 |
| AB-129 | rare | *Stutzerimonas chloritidismutans* strain AW-1 (NR_115115.1) | 1479 | 98.84 |

*Isolates marked with a star might represent novel species as the sequence identity is below the commonly accepted threshold of 98.7 %[1].

**Supplementary Table 2.** Identification of the 23 selected bacterial isolates derived from the cold-water coral specimens from the far site.

| **Strain ID** | **Colour type** | **Closest relative strain by BLAST (Accession number)** | **Sequence length (bp)** | **Sequence Identity (%)** |
| --- | --- | --- | --- | --- |
| AB-33 | white | *Cognaticolwellia mytili* strain RA2-7 (NR_156046.1) | 1483 | 99.66 |
| AB-27 | white | *Pseudovibrio ascidiaceicola* strain NBRC (NR_113916.1) | 1426 | 99.64 |
| AB-12 | white | *Pseudoalteromonas agarivorans* DSM 14585 chromosome I, complete sequence (CP011011.1) | 1467 | 99.86 |
| AB-17 | white | *Pseudoalteromonas agarivorans* DSM 14585 chromosome I, complete sequence (CP011011.1) | 1494 | 99.53 |
| AB-7 | white | *Pseudoalteromonas agarivorans* DSM 14585 chromosome I, complete sequence (CP011011.1) | 1476 | 99.80 |
| AB-1 | white | *Pseudoalteromonas agarivorans* DSM 14585 chromosome I, complete sequence (CP011011.1) | 1475 | 99.46 |
| AB-67* | white | *Pseudoalteromonas arctica* A 37-1-2 chromosome II, complete sequence (CP011026.1), or *Ps. distincta*, or *Ps. nigrifaciens* | 1450 | 99.86 |
| AB-16 | white | *Vibrio splendidus* strain LMG19031 (AP025508.1) | 1473 | 99.52 |
| AB-4 | white | *Vibrio bathopelagicus* strain Sal10 chromosome I, complete sequence (CP062500.1) | 1499 | 99.60 |
| AB-29 | white | *Vibrio syngnathi* strain K08M4 chromosome 1, complete sequence (CP017916.1) or *Vibrio echinoideorum* strain DSM107264 (AP025483.1) | 1497 | 99.73 |
| AB-64 | white | *Vibrio echinoideorum* strain DSM107264 (AP025483.1) | 1470 | 99.86 |
| AB-58 | **orange** | *Kordia aestuariivivens* strain YSTF-M3 (MN872426.1) | 1459 | 98.68 |
| AB-6 | **orange** | *Pseudalkalibacillus hwajinpoensis* strain SW-72 (NR_025264.1) | 1493 | 99.58 |
| AB-9 | **orange** | *Pseudoalteromonas neustonica* strain PAMC 28425 (NR_151996.1) | 1483 | 99.86 |
| AB-19 | **orange** | *Pseudoalteromonas neustonica* strain PAMC 28425 (NR_151996.1) | 1485 | 99.72 |
| AB-2 | **orange** | *Pseudoalteromonas neustonica* strain PAMC 28425 (NR_151996.1) | 1484 | 99.86 |
| AB-30 | **orange** | *Shewanella aestuarii* strain JCM 17801 (MT760375.1) | 1527 | 99.86 |
| AB-70 | **orange** | *Shewanella electrodiphila* strain MAR441 (FR744787.1) | 1485 | 99.59 |
| AB-108* | **orange** | *Shewanella glacialimarina* strain TZS-4 chromosome, complete genome (CP041216.1) | 1505 | 98.61 |
| AB-13 | **orange** | *Shewanella kaireitca* strain c931 (NR_040951.1) | 1483 | 98.85 |
| AB-71 | rare | *Aliivibrio logei* strain 584 (LR813682.1) | 1451 | 99.31 |
| AB-15 | rare | *Pseudoalteromonas ulvae* strain UL12 (NR_025032.1) | 1474 | 98.82 |
| AB-18* | rare | *Pseudoalteromonas ulvae* strain UL12 (NR_025032.1) | 1479 | 98.67 |

*Isolates marked with a star might represent novel species as the sequence identity is below the commonly accepted threshold of 98.7 %.

**Supplementary Table S3.** Types of antibiotics used in the present study and their effects on bacterial cells[2–4]. The first four antibiotics, marked with * represent antibiotics used in the Comau Fjord salmon culture as reported by SERNAPESCA 2021.

| **Antibiotic** | **Class** | **Antibiotic action and target** |
| --- | --- | --- |
| Florfenicol* | Amphenicols | Inhibits ribosomal activity, thereby disrupting bacterial protein synthesis |
| Oxytetracycline* | Tetracyclines | Inhibits protein synthesis in bacteria |
| Tilmicosin* | Macrolides | Inhibits bacterial protein synthesis due to their ability to bind the bacterial 50S ribosomal subunit causing the cessation of bacterial protein synthesis |
| Erythromycin* | Macrolides | Binds to 50S subunit of bacterial rRNA complex, inhibiting protein synthesis |
| Amoxicillin | β-Lactams/ Penicilline | Interferes with peptidoglycan synthesis |
| Ampicillin | β-Lactams/ Aminopenicilline | Inhibits transpeptidation in bacteria, i.e. the cross-linking of cell wall components by transpeptidase, carboxypeptidase, endopeptidase |
| Bacitracin | Polypeptides | Interferes with the function of lipid membrane carrier molecules, which transport building blocks of the peptidoglycan cell wall out of the inner membrane |
| Polymyxin B | Polypeptides | Binds to negatively-charged site in lipopolysaccharide layer, altering outer membrane permeability; fatty acid portion of antibiotic dissolves in hydrophobic region of cytoplasmic membrane, disrupting membrane integrity; leakage of cellular molecules, inhibiting cellular respiration: binds and inactivates endotoxin |
| Streptomycin | Aminoglycoside | Inhibits protein synthesis by binding to 16S rRNA of the 30S ribosomal subunit |
| Kanamycin | Aminoglycoside | Binds to 30S ribosomal subunit, causing incorrect alignment with mRNA, causes non-functional polypeptides |
| Trimethoprim | Diaminopyrimidine | Acts as competitive inhibitors of the enzyme dihydropteroate synthase (DHPS), an enzyme involved in folate synthesis |
| Fosfomycin | Epoxid | Inhibits an enzyme-catalyzed reaction in the first step of the synthesis of the bacterial cell wall |
| Lincomycin | Lincosamide | Binds to 50S subunit of bacterial ribosome, preventing peptide bond formation upon transcription, thus inhibiting protein synthesis |
| Ciprofloxacin | Fluorochinolone | Inhibits DNA gyrase and type II and IV topoisomerase which are necessary in separating DNA, hence inhibiting cell division |
| Metronidazole | Nitroimidazole | Inhibits protein synthesis by interacting with DNA and causing loss of DNA helical structure and strand breaks |


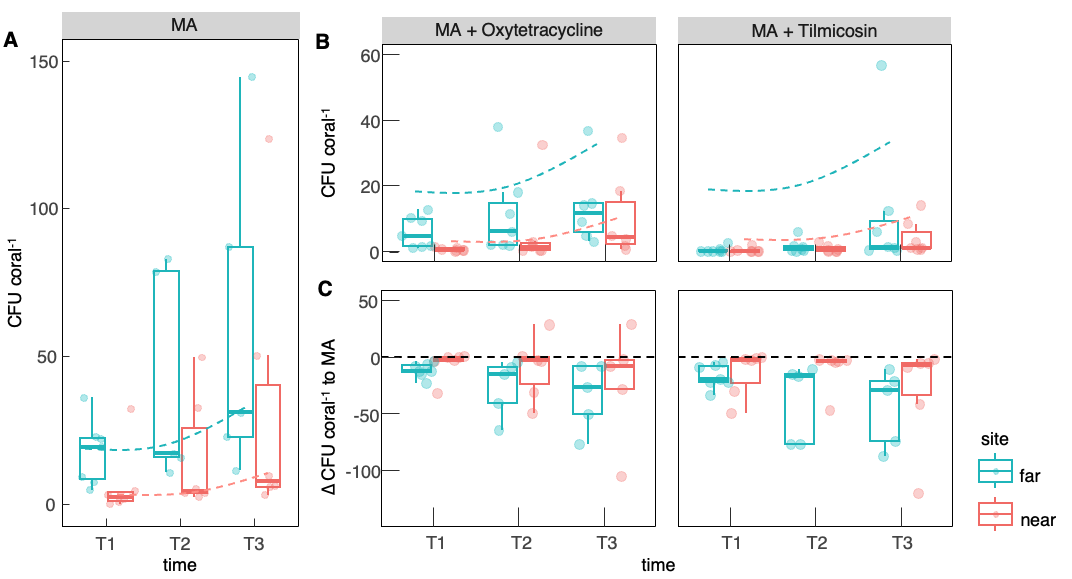


**Supplementary Figure S1. Culturable bacteria from corals collected near and far from salmon farms and cultured on marine agar (MA) and MA supplemented with common salmon farm antibiotics (oxytetracycline and tilmicosin)**. Mean total colony forming units (CFUs) on MA (a) as well as on MA with oxytetracycline (b) and tilmicosin (c) over time (T1 - 3 days, T2 - 7 days and T3 - 35 days) derived from coral specimen (n = 5 - 7) tissue homogenates sampled close (salmon) and far (turquoise) from salmon farm activity. The effect of supplemented antibiotics was quantified as ∆CFU count (d, e) by subtracting mean CFU on MA from the mean counts per coral specimen and time point on MA supplemented with oxytetracycline (d) and with tilmicosin (e).

**References**

1. Chun, J. *et al.* Proposed minimal standards for the use of genome data for the taxonomy of prokaryotes. *International Journal of Systematic and Evolutionary Microbiology* **68**, 461–466 (2018).

2. Trif, E. *et al.* Old Antibiotics Can Learn New Ways: A Systematic Review of Florfenicol Use in Veterinary Medicine and Future Perspectives Using Nanotechnology. *Animals* **13**, 1695 (2023).

3. Butler, M. S. *et al.* A Review of Antibacterial Candidates with New Modes of Action. *ACS Infect. Dis.* **10**, 3440–3474 (2024).

4. Halawa, E. M. *et al.* Antibiotic action and resistance: updated review of mechanisms, spread, influencing factors, and alternative approaches for combating resistance. *Front. Pharmacol.* **14**, (2024).
